# Compact localized states in magnonic Lieb lattices

**DOI:** 10.1038/s41598-023-39816-w

**Published:** 2023-08-04

**Authors:** Grzegorz Centała, Jarosław W. Kłos

**Affiliations:** Institute of Spintronics and Quantum Information, Faculty of Physics, Adam Mickiewicz University, Poznań, Uniwersytetu Poznańskiego 2, 61-614 Poznań, Poland

**Keywords:** Ferromagnetism, Magnetic properties and materials, Spintronics

## Abstract

Lieb lattice is one of the simplest bipartite lattices, where compact localized states (CLS) are observed. This type of localization is induced by the peculiar topology of the unit cell, where the modes are localized only on selected sublattices due to the destructive interference of partial waves. We demonstrate the possibility of magnonic Lieb lattice realization, where flat bands and CLS can be observed in the planar structure of sub-micron in-plane sizes. Using forward volume configuration, the Ga-doped YIG layer with cylindrical inclusions (without Ga content) arranged in a Lieb lattice with 250 nm period was investigated numerically (finite-element method). The structure was tailored to observe, for a lowest magnonic bands, the oscillatory and evanescent spin waves in inclusions and matrix, respectively. Such a design reproduces the Lieb lattice of nodes (inclusions) coupled to each other by the matrix with the CLS in flat bands.

## Introduction

**Figure 1:**
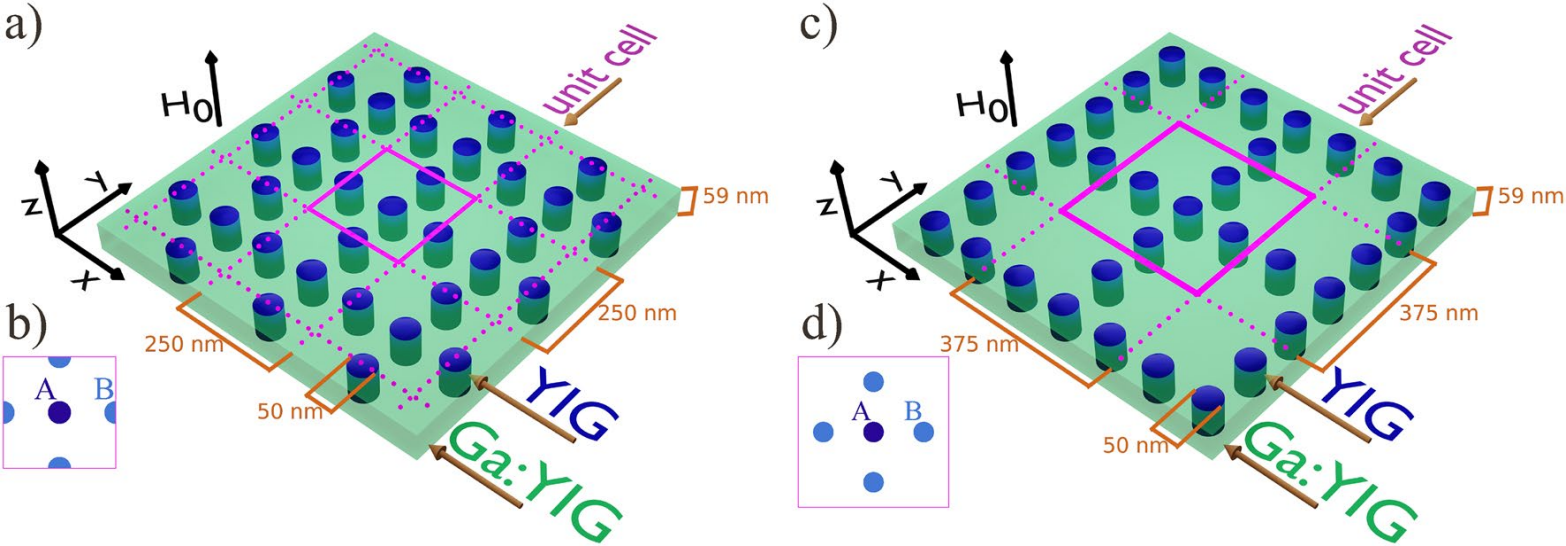
Basic (**a**,**b**) and extended (**c**,**d**) magnonic Lieb lattices. Both planar magnonic structures consist of YIG cylindrical nanoelements embedded within Ga:YIG matrix. Dimensions of the ferromagnetic unit cell for the basic Lieb lattice are equal to 250 $\times$ 250 $\times$ 59 nm whereas for the extended Lieb lattice dimensions are 375 $\times$ 375 $\times$ 59 nm. The unit cell contains three and five inclusions of 50 nm in diameter for basic and extended Lieb lattice (Lieb-5), respectively. In both cases, the separation between centers of inclusions is equal to 125 nm. The structure of basic (**a**) and extended (**c**) Lieb lattice, and top view of basic (**b**) and extended (**d**) Lieb lattice unit cell. Both structures are consisted of one node (inclusion) from minority sublattice *A* and two (four) nodes (inclusions) from majority sublattice for basic Lieb lattice (extended Lieb lattice).

There are many mechanisms leading to wave localization in systems with long-range order, i.e. in crystals or quasicrystals. The most typical of these require (i) the local introduction of defects, including the defects in the form of surfaces or interfaces^1^ (ii) the presence of global disorder^2^, (iii) the presence of external fields^3^ or (iv) the existence of many-body phenomena^4^. However, since at least the late 1980s, it has been known that localization can occur in unperturbed periodic systems in the absence of fields and many-body effects, and is manifested by the presence of flat, i.e., dispersion-free bands in the dispersion relation. The pioneering works are often considered to be the publications of Sutherland^5^ and Lieb^6^, who found the *flat bands* of zero energy^7^ for bipartite lattices with the use of tight-binding model with the hoppings occurring only between sites of different sublattices. The Lieb lattice is regarded as the simplest realization of this type of systems^6,8^. The Lieb lattice is a complex lattice, where the nodes of minority (square) sublattice, connect to each other only via the nodes from other two majority (square) sublattices (Fig. 1a). In the case of extended Lieb lattices^9–11^, the nodes from minority form chains: dimmers, trimmers, etc. (Fig. 1c). An intuitive explanation for the presence of the flat bands is the internal isolation of excitations located in one of the sublattices. The canceling of excitations at one sublattice is the result of destructive interference and local symmetry within the complex unit cell^12^. When only one of the sublattices is excited, the other sublattice does not mediate the coupling between neighboring elementary cells, and the phase difference between the cells is irrelevant to the energy (or the frequency) of the eigenmode on the whole lattice—i.e. the Bloch function. The Bloch functions for flat band are then degenerated for every value of wave number ${\textbf{k}}$. The linear combination of Bloch functions differing in ${\textbf{k}}$ (with a coefficients $f({\textbf{k}})e^{{\textbf{R}}\cdot {\textbf{k}}}$, where $f({\textbf{k}})$ is arbitrary continuous function) are localized around lattice vector ${\textbf{R}}$, similarly like Wannier functions. Such kinds eigenmodes are called *compact localized states* (CLS)^13–17^ and show a certain resistance to the introduction of defects^18,19^. The flat bands with CLS are the platform for the studies of Anderson localization^20^, and unusual properties of electric conductivity^21^. A similar localization is observed in the quasicrystals where the arrangements of the elements composing the structure are replicated aperiodically and self-similarly throughout the system^22,23^. Then, the excitation can be localized on such patterns. The CLS in Lieb lattices have a form of loops (plaquettes) occupying the majority nodes and that kind of states do not form a complete base for the flat band (due to singularity at M point in Brillouin zone). However, the set of CLS can be supplemented by the states occupying only one sublattice of majority nodes. Such states are localized at lines of nodes, and are called *noncontractible loop states* (NLS)^15,16,24^.

The topic of Lieb lattices and other periodic structures with compact localization and flat bands was renewed^8^ about 10 years ago when physical realizations of synthetic Lieb lattices began to be considered for electronic systems^25,26^, optical lattices^27,28^, superconducting systems^29,30^, in phononics^31^, and photonics^15,32^. In a real system, where the interaction cannot be strictly limited to the nearest elements of the structure, the bands are not perfectly flat. Therefore, some authors use the extended definition of the flat band to consider the bands that are flat only along particular directions or in the proximity of high-symmetry Brillouin zone points^33^. In tight-binding models, this effect can be included by considering the hopping to at least next-nearest neighbors^34,35^. Similarly, the crossing of the flat band by Dirac cones can be transformed into anti-crossing and lead to opening of the gaps, separating the flat band from dispersive bands. This effect can be induced by the introduction of spin-orbit term to tight-binding Hamiltonian (manifested by the introduction of Peierls phase factor to the hopping) or by dimerization of the lattice (by alternative changes of hoppings or site energies)^34–38^. The latter scenario can be easily observed in real systems where the position of rods or wells (mimicking the sites of Lieb lattice) and contrast between them can be easily altered^39^. Opening the narrow gap between flat band and dispersive bands for Lieb lattice is also fundamentally interesting because it leads to the appearance of so-called Landau–Zener tunneling^40^.

The isolated and perfectly flat bands for Lieb lattices are topologically trivial—their Chern number is equal to zero^41^. For weakly dispersive (i.e. almost flat) bands the Chern numbers can be different then zero^42^. However, when the flat band is intersected by dispersive bands, then it can exhibit the discontinuity of Hilbert–Schmidt distance between eigenmodes corresponding to the wave vectors just before and just after the crossing. Such an effect is called singular band touching^17^. This limiting value of Hilbert–Schmidt distance is bulk invariant, different from the Chern number.

One of the motivations for the photonic implementation of systems with flat, or actually nearly flat bands^43^, was the desire to reduce the group velocity of light in order to compress light in space, which leads to the concentration of the optical signal and an increase in the light-matter interaction, or the enhancement of non-linear effects. Another, more obvious application is the possibility of realizing delay lines that can buffer the signal to adjust the timing of optical signals^44^. A promising alternative to photonic circuits are magnonic systems, which allow signals of much shorter wavelengths to be processed in devices several orders of magnitude smaller^45,46^. For this reason, it seems natural to seek a magnonic realization of Lieb lattices.

Lieb lattices have also been studied in the context of magnetic properties, mainly due to the possibility of enhancing ferromagnetism in systems of correlated electrons^47^, where the occurrence of flat bands with zero kinetic energy was used to expose the interactions. There are also known single works where the spin waves have been studied in the Heisenberg model in an atomic Lieb lattice, such as the work on the magnon Hall effect^48^. However, the comprehensive studies of spin waves in nanostructures that realize magnonic Lieb lattices and focus on wave effects in a continuous model have not been carried out so far.

## Results

In this paper, we propose the realization of such lattices based on a magnonic structure (Fig. 1a,c) in the form of a perpendicularly magnetized magnetic layer with spatially modulated material parameters or spatially varying static internal field.

We consider planar magnonic crystals^49^ (MCs) to design the magnonic Lieb lattice, owing to the relative ease of fabrication of such structures and their experimental characterization^50–52^. We propose realistic systems that mimic the main features of the tight-binding model of Lieb lattice^17,34^. Investigated MCs consist of yttrium-iron-garnet (YIG) doped with gallium (Ga:YIG) matrix and YIG cylindrical inclusions arranged in Lieb lattice (Fig. 1). Doping YIG with Gallium is a procedure where magnetic ${\text{Fe}}^{3+}$ ions are replaced by non-magnetic ${\text{Ga}}^{3+}$ ions. This method not only decreases saturation magnetization $M_{S}$ but, simultaneously, arises uniaxial out-of-plane anisotropy, that ensures the out-of-plane orientation of static magnetization in Ga:YIG layer at a relatively low external field applied perpendicularly to the layer. Discussed geometry, i.e. forward volume magnetostatic spin-wave configuration, does not introduce an additional anisotropy in the propagation of spin waves related to the orientation of static magnetization.

The design of the Lieb lattice requires the partial localization of spin-wave in inclusions, which can be treated as an approximation of the nodes from the tight-binding model. Furthermore, the neighboring inclusions in the lattice have to be coupled strongly enough to sustain the collective spin-wave dynamics, and weakly enough to minimize the coupling between further neighbors. Therefore, the geometrical and material parameters were selected to ensure the occurrence of oscillatory excitations in the YIG inclusions and exponentially evanescent spin waves in the Ga:YIG matrix. The size of inclusions was chosen small enough to separate the three lowest magnonic bands with almost uniform magnetization precession inside the inclusion from the bands of higher frequency, where the spin waves are quantized inside the inclusions. Also, the thickness of the matrix and inclusion was chosen in a way that there are no nodal lines inside the inclusion. The condition which guarantees the focusing of magnetization dynamics inside the inclusions is fulfilled in the frequency range below the ferromagnetic resonance (FMR) frequency of the out-of-plane magnetized layer made of Ga:YIG (matrix material): $f_\mathrm{FMR,Ga:YIG}=~$4.95 GHz and above the FMR frequency of out-of-plane magnetized layer made of YIG (inclusions material): $f_{\text{FMR,YIG}}=2.42$ GHz. These limiting values were obtained using the Kittel formula for out-of-plane magnetized film: $f_{\text{FMR}}=(|\gamma| /2\pi )|{\mu _0H_{0}+\mu _0H_{\text{ani}}-\mu _0M_{S}|}$, where we used the following values of material parameters^53^ for YIG: gyromagnetic ratio $|\gamma| = 177$ rad T$^{-1}$ns$^{-1}$, magnetization saturation $\mu _{0}M_{ S} = 182.4$ mT, exchange stiffness constant $A = 3.68$ pJ m$^{-1}$, (first-order) uniaxial anisotropy field $\mu _{0}H_{\text{ani}} = -3.5~$mT, and for Ga:YiG: $|\gamma| = 179$ rad T$^{-1}$ns$^{-1}$, $\mu _{0}M_{ S} = 20.2$ mT, $A = 1.37$ pJ m$^{-1}$, $\mu _{0}H_{\text{ani}} = 94.1$ mT. Due to the greatest impact of the first-order uniaxial anisotropy field ($\mu _{0}H_{\text{ani}}$), we decided to neglect higher-order terms of uniaxial anisotropy and cubic anisotropy of both YIG and Ga:YIG. Due to the presence of out-of-plane anisotropy and relatively low saturation magnetization, we could consider a small external magnetic field $\mu _{0}H_{0} = 100$ mT to reach saturation state. It is worth noticing that without the evanescent spin waves in the ferromagnetic matrix, the appropriate coupling between inclusions would not be possible. Therefore, the realization of the Lieb lattice in form of the array of ferromagnetic nanoelemets embedded in air/vacuum seems to be very challenging (see the exemplary results in Supplementary Note 3).

We also tested the possibility of other realizations of magnonic Lieb lattices. One solution seemed to be the design of a structure in which the concentration of the spin-wave amplitude in the Lieb lattice nodes would be achieved through an appropriately-shaped profile of the static demagnetizing field (see Supplementary Note 2). However, the obtained results were not as promising as for YIG/Ga:YIG system. In the following part of the manuscript, we present the results for the basic Lieb lattice (showed in Fig. 1a,b) and extended Lieb-5 lattice (showed in Fig. 1c,d), based on YIG/Ga:YIG structures. The further extension of the Lieb lattice may be realized by increasing the number of *B* nodes between neighboring *A* nodes. Supplementary Note 1 presents the results for Lieb-7 lattice, where for each site (inclusion) from minority sublattice *A*, we have six nodes (inclusions), grouped in three-element chains, from majority sublattices *B*.

The tight-binding model of the basic Lieb lattice with hopping restricted to next-neighbors gives three bands in the dispersion relation. The top and bottom bands are symmetric with respect to the second, perfectly flat band, and intersect with this dispersionless band at M point of 1$^{\text{st}}$ Brillouin zone, with constant slope forming two Dirac cones^8,28^. In a realistic magnonic system, the spin-wave spectrum showing the particle-hole symmetry with a zero-energy flat band is difficult to reproduce because (i) the dipolarly-dominated spin waves, propagating in magnetic film, experience a significant reduction of the group velocity with an increase of the wave vector (this tendency is reversed for much larger wave vectors were the exchange interaction starts to dominate)^54^, (ii) the dipolar interaction is long-range. The first effect makes the lowest band wider than the third band, and the latter one induces the finite width of the second band^34^. We are going to show that this weakly dispersive band supports the existence of CLS. Therefore, we will still refer to it as *flat band*, which is a common practice for different kinds of realization of Lieb lattices in electronics^26^, photonics^55^ or optical lattices^28^.

**Figure 2:**
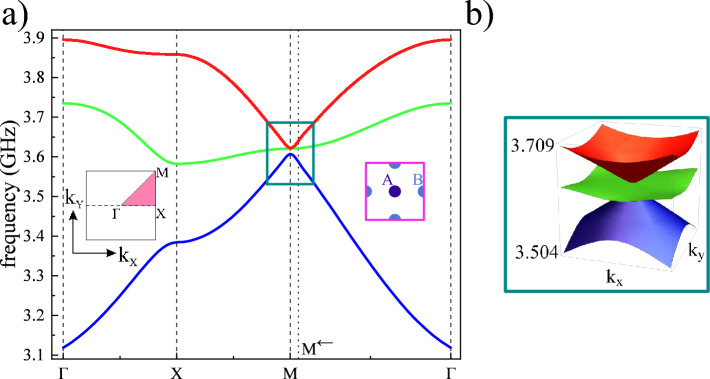
Dispersion relation for the basic magnonic Lieb lattice containing three inclusions in the unit cell: one inclusion *A* from minority sublattice and two inclusions *B* from majority sublattices (see Fig. 1a,b). (**a**) The dispersion relation is plotted along the high-symmetry path $\Gamma$-X-M-$\Gamma$ (see the inset). The lowest band (blue) and the highest band (red) create Dirac cones almost touching (**b**) in the M point. The middle band (green) is relatively flat in the vicinity of the M point.

The results obtained for the basic magnonic Lieb lattice (which is presented in Fig. 1a), are shown in Fig. 2. As we predicted, three lowest bands form a band structure that is similar to the dispersion relation known from the tight-binding model^11^. However, in a considered realistic system there is an infinite number of higher bands, not shown in Fig. 2a. For higher bands, spin waves can propagate in an oscillatory manner in the matrix, hence the system does not mimic the Lieb lattice where the excitations should be associated with the nodes (inclusions) of the lattice.

Due to the fourfold symmetry of the system, the dispersion relation could be inspected along the high-symmetry path $\Gamma$-X-M-$\Gamma$. Frequencies of the first three bands are in the range $f_{\text{FMR,YIG}}-f_\mathrm{FMR,Ga:YIG}$. Their total width is about 0.78 GHz. The first and third band form Dirac cones at M point, separated by a tiny gap of about 15 MHz. The possible mechanism responsible for opening the gap is a small difference in the demagnetizing field in the areas of inclusions *A* (from the minority lattice) and inclusions *B* (from two majority sublattices)—see Supplementary Note 4. Inclusions *A* (*B*) have four (two) neighbors of type *B* (*A*). Although inclusions *A* and *B* have the same size and are made of the same material, the static field of demagnetization inside them differs slightly due to the different vicinity. This effect is equivalent to the dimerization of the Lieb lattice by varying the energy of the nodes in the tight-binding model, which leads to the opening of a gap between Dirac cones and parabolic flattening of them in very close proximity to the M point. It is worth noting that in the investigated system, the gap opens between the first and second bands, while the second and third bands remain degenerated at point M, with numerical accuracy.

**Figure 3:**
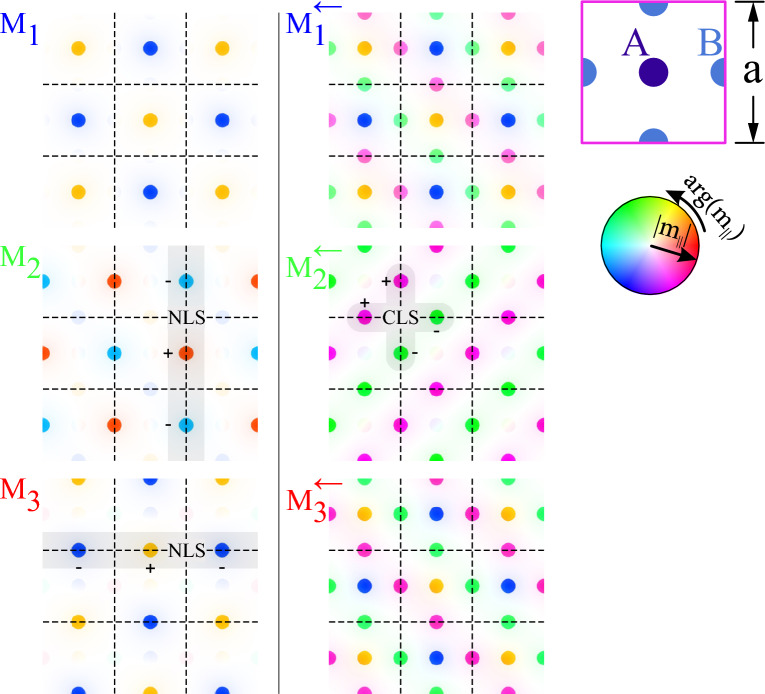
The profiles of the Bloch functions obtained for the basic magnonic Lieb lattice, composed of three inclusions in the unit cell (see Fig. 1a,b). *a*—denotes lattice constant. The modes are presented for each band exactly at M (left column) and in its proximity (M$^\leftarrow$) on the path M-$\Gamma$ (right column). In the presented profiles, the saturation and the color denote the amplitude and phase of the dynamic in-plane component of magnetization. The patterns characteristic for compact localized states (CLS) are presented at the point M$^\leftarrow$ for the second band (right column). The CLS do not occupy minority sublattice *A*. The inclusions *B*, in which the magnetization dynamics is focused, are quite well isolated from each other. One can easily notice that the lattice is decorated by loops (marked by gray patches) where the phase of the precessing magnetization flips between inclusions ($+$ and − signs). Exactly at point M (left column), we observe the degeneracy of the second and third bands. The spin waves occupy *B* inclusions only in one majority sublattice, i.e. along vertical or horizontal lines, flipping the phase from inclusion to inclusion which gives the pattern characteristic to noncontractible loop states (NLS) marked by gray stripes.

The middle band can be described as weakly dispersive. The band is more flat on the X-M path and, in particular, in the vicinity of M point—see Fig. 2b. The small width of the second band can be attributed to long-range dipolar interactions which govern the magnetization dynamics in a considered range of sizes and wave vectors. It is known that even the extension of the range of interactions to next-nearest neighbors in the tight-binding model induces the finite width of the flat band for the Lieb lattice.

To prove that the second band supports the CLS regardless of its finite width, we plotted the profiles of spin-wave eigenmodes (Bloch functions) at M point and in its close vicinity. The results are presented in Fig. 3. The profiles were shown for infinite lattice and are presented in the form of square arrays containing 3 $\times$ 3 unit cells, where the dashed lines mark their edges. It is visible that spin waves are concentrated in the cylindrical inclusions, where the amplitude and phase of precession is quite homogeneous. In calculations, we used the Bloch boundary conditions applied for a single unit cell, which means that at M point the Bloch function is flipped after translation by lattice period, in both principal directions of the lattice and we will not see the single closed loops of CLS or lines of NLS. Exactly at M point, all three bands have zero group velocity. Therefore, the corresponding modes (left column) are not propagating. The lowest band (M$_1$) occupies only inclusions *A* from the minority sublattice where the static demagnetizing field is slightly lower than inside inclusions *B* (see Supplementary Note 4), which justifies its lower frequency and lifting the degeneracy with two higher modes M$_2$ and M$_3$ of the same frequency. Each of the modes M$_2$ and M$_3$ occupy only one of two sublattices *B*, therefore they can be interpreted as NLS. To observe the pattern typical for CLS, we need to move slightly away from M point. The first and third modes have then linear dispersion with high group velocity and the second band remains flat. We selected the point M$^\leftarrow$ shifted from M point toward $\Gamma$ point by 5% of M-$\Gamma$ distance (right column). We can see that the first and third modes M$^\leftarrow _1$, M$^\leftarrow _3$ occupy now all inclusions and the mode M$^\leftarrow _2$ from the flat band has a profile typical for CLS, predicted by tight-binding models:^8,10,11,56,57^:

1 $$\begin{aligned} \vert m_{\textbf {k}}\rangle =\big [\underbrace{-\text {cos}(k_{y}a/2)}_{B_x},\underbrace{0}_A,\underbrace{\text {cos}(k_{x}a/2)}_{B_y}\big ]^{\text {T}}, \end{aligned}$$

where $\vert m_{{\textbf {k}}}\rangle$ is the set of complex amplitudes of the Bloch function in the base of unit cell, i.e. on one inclusion *A* from minority sublattice and two inclusions *B* from majority sublattices ($B_x$—shifted by $(a/2)\hat{{\textbf{x}}}$ form site *A* and $B_y$—shifted by $(a/2)\hat{{\textbf{y}}}$ form site *A*). The symbol ${\textbf {k}}=[k_x,k_y]$ denotes wave vector. From Eq. (1) we can see that (i) CLS (constructed from Bloch functions) will do occupy the minority nodes *A* and (ii) close to M point the phases at two nodes *B*, from different majority sublattices, are opposite. These two features are reproduced for M$^\leftarrow _2$ mode in investigated magnonic Lieb lattice. In the profile of this mode, we marked (by a gray patch) the elementary loop of CLS which is easily identified in finite systems. Here, in an infinite lattice with Bloch boundary conditions, the loops are infinitely replicated with $\pi$ phase shift after each translation *x*- and *y*-direction. The localization at the inclusions *B* and the absence of the spin-wave dynamics in inclusions *A* is observed regardless of the wave vector. Therefore, the coupling can take place only between the next neighbors (inclusions *B*), i.e. on larger distances and mostly due to dipolar interactions, that makes the second band not perfectly flat. It is worth noting that eigenmode (1) corresponds to the “zero-energy mode” ($\Delta \omega =\omega -\omega _0=0$) of the magnonic eigenvalue problem in the NN tight-binding formulation (see Supplementary Note 5):

2 $$\begin{aligned} \Delta \omega \left( \begin{array}{c} m_A\\ m_{B_x}\\ m_{B_y}\\ \end{array} \right) = -\kappa \omega _M \left( \begin{array}{c c c} 0&{}\phi _x({\textbf{k}})&{} \phi _y({\textbf{k}})\\ \phi _x^*({\textbf{k}})&{}0&{}0\\ \phi _y^*({\textbf{k}})&{}0&{}0\\ \end{array} \right) \left( \begin{array}{c} m_A\\ m_{B_x}\\ m_{B_y}\\ \end{array} \right) . \end{aligned}$$

The symbol $\omega _0$ is the (angular) frequency of spin wave mode in a single inclusion embedded in the matrix, or the degenerated frequency of the Lieb lattice in the limit of infinitely large lattice constant $a\rightarrow \infty$, where coupling between NN inclusions go to zero $\kappa \rightarrow 0$. The parameter $\omega _M$ is the magnetization saturation for the inclusions $M_{\text{S}}$, expressed in the units of angular frequency: $\omega _{M}=|\gamma |\mu _0 M_{\text{S}}$. The phase factors: $\phi _x({\textbf{k}})=\phi _x^*({\textbf{k}})=2\text {cos}(k_x a/2)$ and $\phi _y({\textbf{k}})=\phi _y^*({\textbf{k}})=2\text {cos}(k_y a/2)$ are related to the hoppings between NN inclusions in $x-$ and $y-$direction, respectively. It can be also show that the first and third eigenmode have the eigenfrequencies which depends linearly on ${\textbf{k}}$, in the vicinity of M point (${\textbf{k}}=[\pi /a,\pi /a])$ and form the Dirac cone: $\omega ({\textbf{k}})=\omega _0\pm |\kappa \omega _M |a\big |{\textbf{k}}-[\pi/a ,\pi/a ]\big |$—see Fig. 2b for illustration and Supplementary Note 5 for derivation.

**Figure 4:**
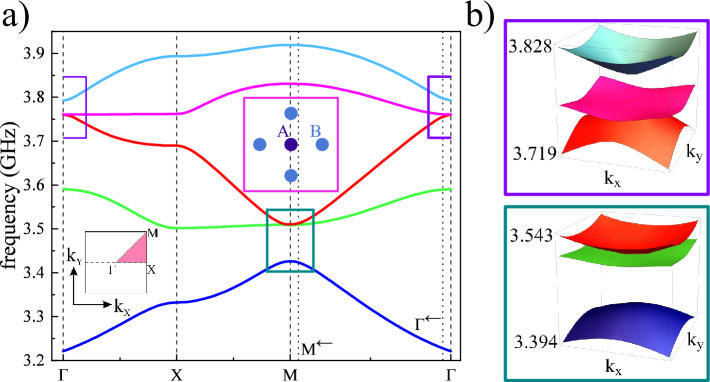
Dispersion relation for the extended magnonic Lieb lattice Lieb-5 containing five inclusions in the unit cell: one inclusion *A* from minority sublattice and four inclusions *B* from majority sublattices (see Fig. 1c,d). (**a**) The dispersion relation is plotted along the high-symmetry path $\Gamma$-X-M-$\Gamma$ (see the inset). The first, third, and fifth bands (dark blue, red, and cyan) are strongly dispersive bands, while the second and fourth bands (green and magenta) are less dispersive and related to the presence of CLS. The system does not support the appearance of Dirac cones, even in case when the interaction is fictitiously limited only to inclusions, according to the tight-binding model. (**b**) The zoomed regions in the vicinity of $\Gamma$ (in a dark green frame) and M points show the essential gaps with relatively low parabolic-like curvatures for top and bottom bands.

Let us discuss now the presence of flat bands and CLS in an extended magnonic Lieb lattice (Lieb-5), containing five inclusions in the unit cell: one inclusion *A* form minority sublattice and four inclusions *B* from majority sublattices, as it is presented in Fig. 1c,d. In the considered structure, we add two additional inclusions *B* into the unit cell in such a way that neighboring inclusions *A* are linked by the doublets of inclusions *B*. The sizes of inclusions, distances between them, the thickness of the layer, and the material composition of the structure remained the same as for the basic Lieb lattice, discussed earlier (Fig. 1a,b).

The dispersion relation obtained for the magnonic Lieb-5 lattice can be found in Fig. 4a. The properties of the extended Lieb lattices are well described in the literature^11,58–60^. The tight-binding model description of Lieb-5 lattices, with information about their dispersion relation and the profiles of the eigenmodes, are presented in numerous papers^11,17,56^. Therefore, it is possible to compare the obtained results with the theoretical predictions of the tight-binding model.

**Figure 5:**
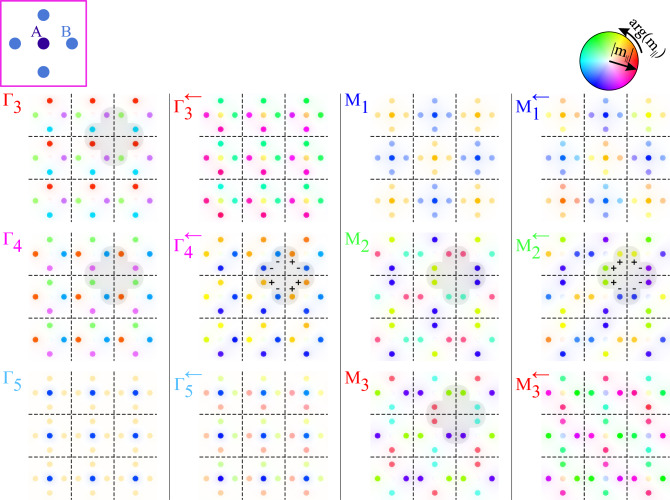
The profiles of Bloch functions obtained for the extended Lieb lattice consisted of 5 inclusions in the unit cell. The modes are presented for bands no. 3–5 in $\Gamma$ point and its proximity $\Gamma ^{\leftarrow }$ (the first and second column). In the third and fourth columns, we presented the profiles for bands no. 1–3 at M point and its vicinity M$^{\leftarrow }$. Each profile of eigenmode is presented on a grid composed of 3 $\times$ 3 unit cells—dashed lines mark the edges of unit cells. The scheme of the unit cell is presented in top-left corner. Exactly at $\Gamma$ (and M) point the bands no. 3 and 4 (no. 2 and 3) are degenerated and profiles: $\Gamma _3$ and $\Gamma _4$ (M$_2$ and M$_3$) have non-standard (for CLS) complementary form—i.e. their combinations $\Gamma _3\pm i\Gamma _4$ (M$_2\pm i$M$_3$) gives NLS. To obtain proper profiles of CLS, where the phase of procession flips around CLS loop, we need to explore the vicinity of $\Gamma$ (M) point—see the gray patches for the mode $\Gamma ^{\leftarrow }_4$ (M$^{\leftarrow }_2$) with $+$ and − signs.

The tight-binding model of Lieb-5 lattice predicts two flat bands with CLS: the second (green) and fourth (magenta) band in the spectrum. The flat bands in the tight-binding model are not intersected by Dirac cones but they are degenerated at $\Gamma$ and M point with the third band (red). These features are reproduced in the investigated magnonic Lieb-5 lattice (Fig. 1c,d). The dispersion relation for this system is presented in the Fig. 4a. Also, we have marked, with two rectangles (dark green and violet), the vicinities of $\Gamma$ and M points, where the flat bands (the fourth and second bands) become degenerated with the third, dispersive band—Fig. 4b. It is easy to notice the essential frequency gaps ($\approx 33$ MHz and $\approx 84~$MHz at $\Gamma$ and M points, respectively), which qualitatively corresponds to the prediction of the tight-binding model. It is worth noting that although the low dispersion bands (the second and fourth band) are in general not perfectly flat, around $\Gamma$ and M points the bands are flattened and the $\Gamma$-X and X-M sections are very flat for the fourth and second band, respectively.

The spin-wave profiles of Bloch functions at the high-symmetry points: $\Gamma$ and M, exhibiting the CLS patterns, are presented in Fig. 5. Exactly at $\Gamma$ and M (the first and third column), we can see the pairs of degenerated mods $\Gamma _3$, $\Gamma _4$ and M$_2$, M$_3$ which exhibit features of CLS predicted by the tight-binding model (see the loops of sites on gray patches): (i) modes occupy only the inclusions *B* from majority sublattices, (ii) doublets of inclusions *B* in the loops of CLS have opposite (the same) phases at $\Gamma$ (M) point. The significant difference is that once we switch one to another *B*-*B* doublet, circulating the CLS loop the phase of precession charges by $\pm \pi /2$ not by 0 or $\pi$. However, when we make combinations of degenerated modes: $\Gamma _3\pm i\Gamma _4$ or M$_2\pm i$M$_3$, then we obtain the NLS occupying the horizontal or vertical lines, where the precession at excited *B* inclusion will be in- or out-of-phase. The patterns of CLS modes are clearly visible when we move slightly away from the high-symmetry point where the degeneracy occurs. In the proximity of $\Gamma$ and M point, one can see the CLS modes for Bloch functions $\Gamma ^{\leftarrow }_4$ and M$^{\leftarrow }_2$ for which the phase of precession takes the relative values close to 0 or $\pi$. The small discrepancies are visible as a slight change in the colors representing the phase, resulting from the fact that we are not exactly in high-symmetry points but shifted by 5% on the path $\Gamma$-M.

The extension of the presented analysis to magnonic Lieb-7 lattice, where the inclusions *A* are linked by the chains composed of three inclusions *B*, is presented in Supplementary Note 1.

## Discussion

We proposed a possible realization of the magnonic Lieb lattices where the compact localized spin-wave modes can be observed in flat bands. The presented system qualitatively reproduces the spectral properties and the localization features of the modes, predicted by the tight-binding model and observed for photonic and electronic counterparts. The magnonic platform for the experimental studies of Lieb lattices seems to be attractive due to the larger flexibility in designing magnonic systems and the steering of its magnetic configuration by external biases. The idea of the magnonic Lieb lattices allows considering many problems related to dynamics, localization, and interactions in flat-band systems taking the advantage of the magnonic systems: presence and possibility of tailoring of long-range interactions, intrinsic non-linearity, etc.

## Methods

The spectra of spin waves and the spatial profiles of their eigenmodes were obtained numerically in a semi-classical model, where the dynamics of magnetization vector ${\textbf {M}}({\textbf {r}},t)$ is described by the Landau–Lifshitz equation^54^:

3 $$\begin{aligned} \frac{d{\textbf {M}}}{dt}=-|\gamma| \mu _{0}[ {\textbf {M}} \times {\textbf {H}}_{\text{eff}} + \frac{\alpha }{M_{S}} {\textbf {M}} \times ({\textbf {M}} \times {\textbf {H}}_{\text{eff}})]. \end{aligned}$$

The symbol ${\textbf {H}}_{\text{eff}}({\textbf {r}},t)$ denotes effective magnetic field.

In numerical calculations, we neglected the damping term since $\alpha$ is small both for YIG and for YIG with Fe substituted partially by Ga (for $\alpha _{\mathrm{Ga:YIG}} = 6.1\times 10^{-4}$ and $\alpha _{\text{YIG}} = 1.3\times 10^{-4}$^53^). The effective magnetic field $H_{\text{eff}}({\textbf{r}},t)$ is calculated as functional derivative of the free energy density $F({\textbf{r}},t)$ with respect to the magnetization ${\textbf{M}}({\textbf{r}},t)$:

4 $$\begin{aligned} H_{\textbf{eff}}({\textbf{r}},t)=-\frac{1}{\mu _0}\frac{\delta F({\textbf{r}},t)}{\delta {\textbf{M}}({\textbf{r}},t)}, \end{aligned}$$

and contains the following components (related to the corresponding terms of the $F({\textbf{r}},t)$): the external field $H_{0}$ (Zeeman energy), exchange field $H_{\text{ex}}$ (energy of exchange interactions), bulk uniaxial anisotropy field $H_{\text{ani}}$ (energy of uniaxial magnetocrystalline anisotropy) and dipolar field $H_{\text{d}}$ (energy of dipolar interactions):

5 $$\begin{aligned} {\textbf {H}}_{\text{eff}}({\textbf {r}},t)=H_{0}\,\hat{{\textbf {z}}}+\frac{2A}{\mu _{0}M^{2}_{S}}\Delta {\textbf {M}}({\textbf {r}},t)+H_{\text{ani}}({\textbf {r}})\,\hat{{\textbf {z}}}+{\textbf {H}}_{\text{d}}({\textbf {r}},t), \end{aligned}$$

where the *z*-direction is normal to the plane of the magnonic crystal. We assume that the sample is saturated in *z*-direction and magnetization vector precesses around this direction. The material parameters ($M_{S}$, *A* and $\gamma$) are constant within matrix and inclusions. Using the magnetostatic approximation, the dipolar term of the effective magnetic field may be expressed as a gradient of magnetic scalar potential^54^:

6 $$\begin{aligned} {\textbf {H}}_{\text{d}}({\textbf {r}},t)=-\nabla \varphi ({\textbf {r}},t) \end{aligned}$$

By using the Gauss equation, magnetic scalar potential may be associated with magnetization as follows:

7 $$\begin{aligned} \Delta \varphi ({\textbf {r}},t) = \nabla \cdot {\textbf {M}}({\textbf {r}},t) \end{aligned}$$

We used the COMSOL Multiphysics^61^ to implement the Landau–Lifshitz equation Eq. (3) and performed finite element method computation for the defined geometry of magnonic Lieb lattices. All the equations were implemented in the Mathematics module which contains different forms of partial differential equations. To obtain the static demagnetization field we solved Eq. (7) using stationary study. Then using the eigenfrequency study for each wave vector we solved Eq. (7) (obtaining a dynamic demagnetization field) and then Eq. (3) (solving the Landau–Lifshitz equation). To obtain free decay of magnetic scalar potential in the model, we applied $5~\upmu$m of a vacuum above and underneath the structure. At the bottom and top surface of the model with vacuum, we applied the Dirichlet boundary condition for zeroing of magnetic scalar potential. We use the Bloch theorem for each variable (magnetic scalar potential and components of magnetization vector) at the lateral surfaces of a unit cell. We selected the symmetric unit cell with minority node *A* in the centers to generate a symmetric mesh that does not perturb the four-fold symmetry of the system. This approach is of particular importance for the reproduction of the eigenmodes profiles in high-symmetry points. In our numerical studies, we used 2D wave vector ${\textbf {k}}=k_{x}\hat{{\textbf {x}}}+k_{y}\hat{{\textbf {y}}}$ as a parameter for eigenvalue problem which was selected along the high-symmetry path $\Gamma$-X-M-$\Gamma$ to plot the dispersion relation.

### Supplementary Information

Supplementary Information.

## Data Availability

The datasets used and analysed during the current study available from the corresponding author on reasonable request.
