# Supplementary material for: Compact localized states in magnonic Lieb lattices
Source: Sci Rep. 2023 Aug 4;13:12676. doi: 10.1038/s41598-023-39816-w (PMC10403553; doi:10.1038/s41598-023-39816-w)
Supplement: Supplementary file 1 — Supplementary Information. [file 41598_2023_39816_MOESM1_ESM.pdf]

Supplementary Information:
Compact localised states in magnonic Lieb lattices

Grzegorz Centała and Jarosław W. Kłos*

Institute of Spintronics and Quantum Information,

Faculty of Physics, Adam Mickiewicz University, Poznań,

Uniwersytetu Poznańskiego 2, Poznań 61-614, Poland

Supplementary Note 1. Doubly-extended Lieb lattice

We can generate further extensions of the magnonic Lieb lattice [1] by adding more inclusions B, i.e. by introducing additional majority sublattices. We consider here a doubly-extended Lieb lattice (Lieb-7) to check to what extent the magnonic system corresponds to the tight-binding model. The mentioned lattice consists of seven nodes; six belong to majority sublattices B and one belongs to minority sublattice A (Supplementary Fig. 1). The magnetic parameters were kept as for basic and Lieb-5 lattices, considered in the manuscript. The geometrical parameters have changed only as a result of the introduction of additional inclusions B. Therefore, the unit cell has increased to the size of 500x500 nm.

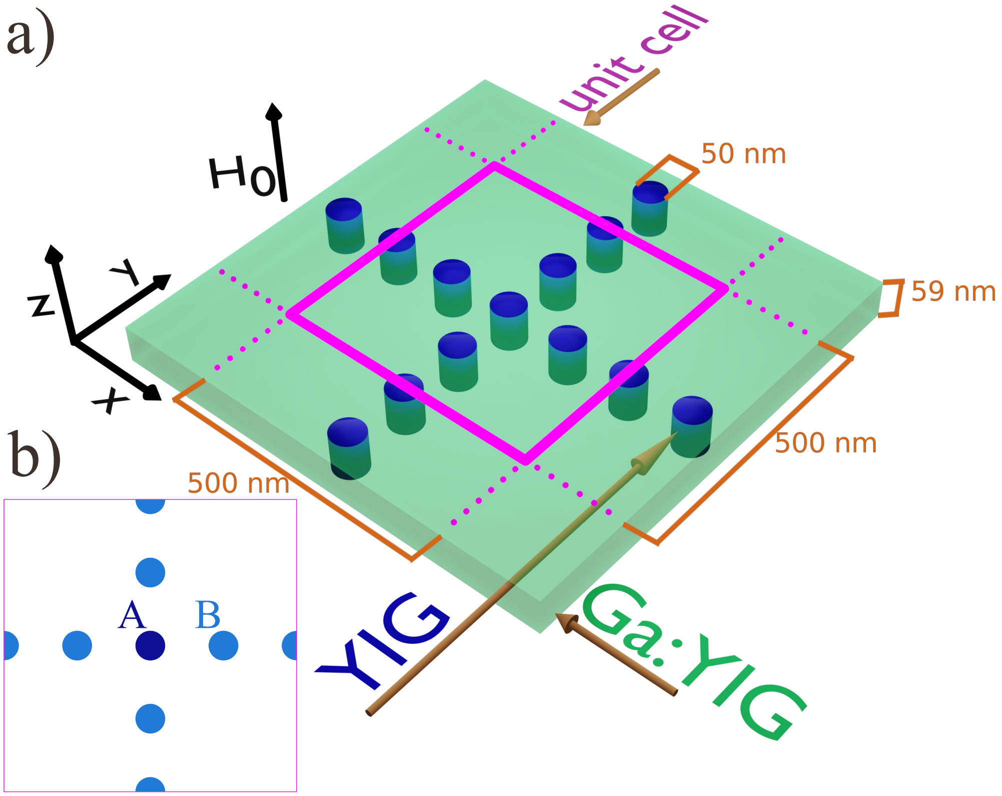

Supplementary Figure 1. Doubly-extended magnonic Lieb lattice: Lieb-7. Dimensions of the ferromagnetic unit cell are equal to 500x500x59 nm. The unit cell contains seven inclusions of 50 nm diameter. (a) The structure of extended Lieb lattice, and (b) top view on Lieb-7 lattice unit cell where the node (inclusion) from minority sublattice A and two nodes (inclusions) from two majority sublattices B are marked.

* klos@amu.edu.pl

In the case of a doubly-extended Lieb lattice (Lieb-7), we expect (according to the work [2, 3]) to obtain seven bands in the dispersion relation. The tight-binding model predicts

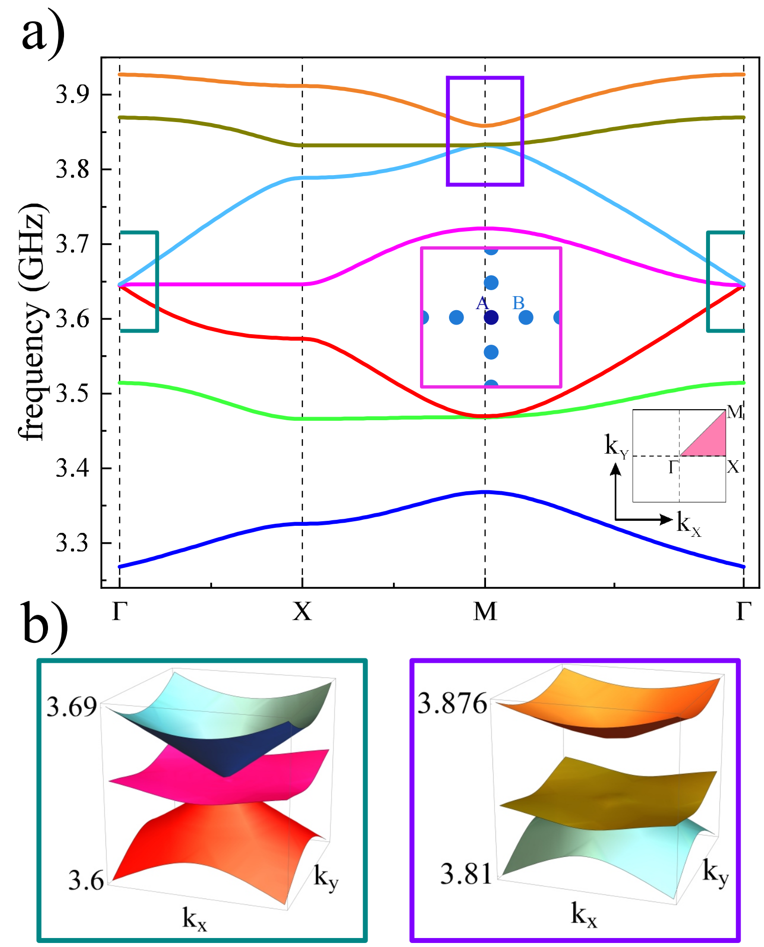

Supplementary Figure 2. Dispersion relation for the doubly-extended magnonic Lieb lattice (Lieb-7) containing seven inclusions in the unit cell: one inclusion A from minority sublattice and six inclusions B from majority sublattices (Supplementary Fig. 1). (a) The dispersion relation is plotted along the high-symmetry path Γ-X-M-Γ (see the inset). The first, third, fifth, and seven bands (dark blue, red, cyan, and orange) are dispersive, while the second, fourth, and sixth bands (green, magenta, and dark green bands) are the flatter bands, supporting the magnonic CLS. Dirac cones occur at the Γ point and almost interact with the flatter fourth band, while at M point, we observe the degeneracy of the dispersive parabolic third (fifth) band with a flatter second (six) band. (b) The zoomed vicinity of Γ point (dark green frame) and M point (violet frame) regions are presented in 3D.

that the bands will be symmetric with respect to the fourth band, exhibiting particle-hole symmetry. However, due to the dipolar interaction, we did not expect such symmetry. Another feature that one may deduce from the tight-binding model is that bands no. 2, 4, and, 6 should be flat while bands no. 1, 3, 5, and, 7 are considered dispersive. Moreover, band no. 3 and, 5 suppose to form a Dirac cone intersecting flat band no. 4 at the Γ point.

We calculated the dispersion relation for magnonic Lieb-7 lattice (Supplementary Fig. 2(a)), which share many properties with those characteristic for the tight-binding model [3]: (i) third and fifth bands form the Dirac cones which almost intersect the flatter fourth band at Γ point; (ii) the third (and fifth) band has a parabolic shape at M point where it is degenerated with the second (and six) band which is weakly dispersive. The mentioned regions of dispersion are presented as 3D plots in Supplementary Fig. 2(b). Also, we are going to discuss shortly the profiles of spin-wave eigenmodes (including CLS) in these two regions of the dispersion relation, which are presented in Supplementary Fig. 3.

Dirac cones appear at the Γ point for bands no. 3 and 5. At this point, as for the basic magnonic Lieb lattice (see Fig. 2 in the main part of the manuscript) there is a very narrow gap of the width of about 2 MHz. The profiles Γ_4 and Γ_5 (left column in Supplementary Fig. 3) represent the degenerated states originating from flat and dispersive bands. Both of them do not occupy the inclusions A and are more focused on two inclusions B arranged in horizontal (Γ_4) and vertical lines (Γ_5) – see gray stripes. Therefore, their profiles are similar to NLS, where the first and third inclusion B in each three-element chain, linking inclusions A, precesses out-of-phase and the second (central) inclusion B remains unoccupied.

At the M point, the M_5 and M_6 bands are degenerated. For these bands, the spin waves are localized in all inclusions B and do not occupy inclusions A (see right column of Supplementary Fig. 3) The first and third inclusion B in each three-element chain, linking inclusions A, precess in-phase, whereas the second (central) inclusion B precesses out-of-phase with respect to the first and third one. This pattern of occupation of inclusions and the phase relations between them is similar to one observed for CLS (see gray patches marking the loops of inclusions in the left column of Supplementary Fig. 3), but has one significant difference. The phase difference between successive three-element chains of inclusions B, in the loop, is equal to $\pm\pi/2$. However, the linear combination of the modes $\mathrm{M}_5 \pm i\mathrm{M}_6$ produces, similarly to the case of the Lieb-5 lattice, the NLS. To observe the proper profiles

of CLS or NLS, we need to shift slightly from the high-symmetry points Γ and M to cancel the degeneracy.

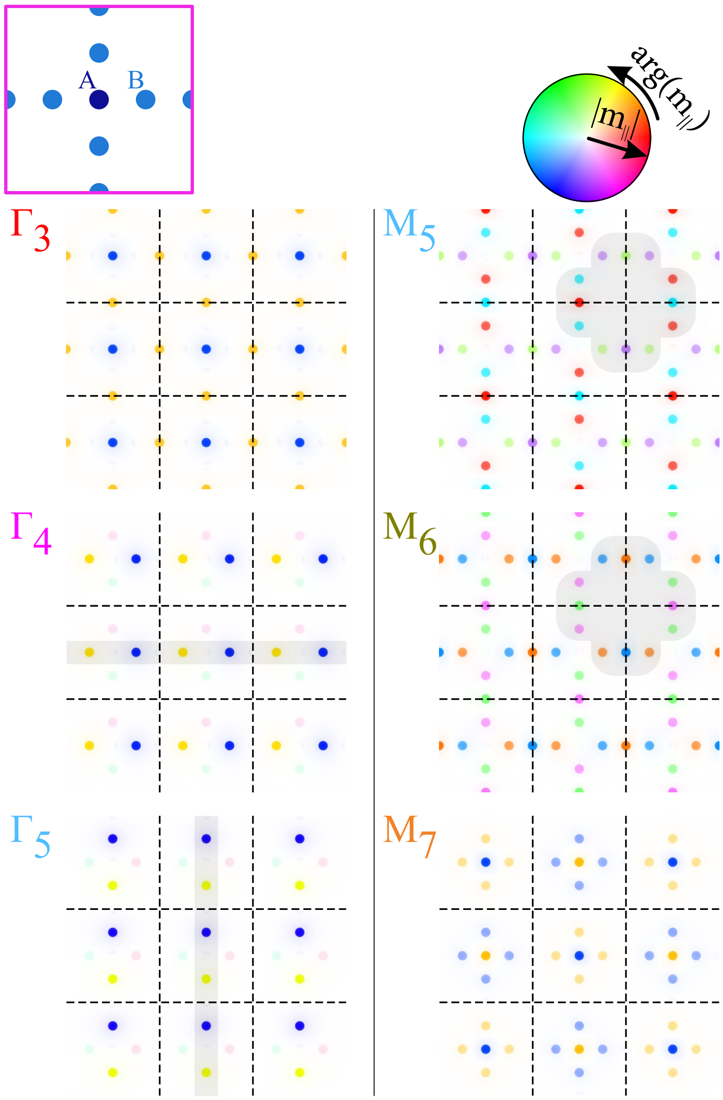

Supplementary Figure 3. The profiles of eigenmodes were obtained for magnonic Lieb-7. The modes are presented for bands no. 3-5 at Γ point and 5-7 at M point. The modes denoted as Γ_3 and Γ_4 are degenerated whereas the Γ_5 is separated from them by extremely small gap of about 2 MHz. At M point, we show the profiles for bands no. 5, 6, and 7. The modes M_5 and M_6 are degenerated and separated from M_7 by essential gap – predicted by the tight-binding model.

Supplementary Note 2. Realization of Lieb lattice by shaping demagnetizing field

We have considered also an alternative realization method for a magnonic Lieb lattice in a ferromagnetic layer. This approach is based on shaping the internal demagnetizing field. The structure under consideration is presented in Supplementary Fig. 4. It consists of a thin (14.75 nm) and infinite CoFeB layer on which a Py antidot lattice (ADL), of 14.75 nm thickness, is deposited.

The cylindrical holes in ADL are arranged in the shape of the basic Lieb lattice. The size of the unit cell and diameter of holes remains the same as for the basic Lieb lattice proposed in the main part of the manuscript (see Fig. 1(a)). Due to the absence of perpendicular magnetic anisotropy (PMA), we decided to apply a much larger external magnetic field

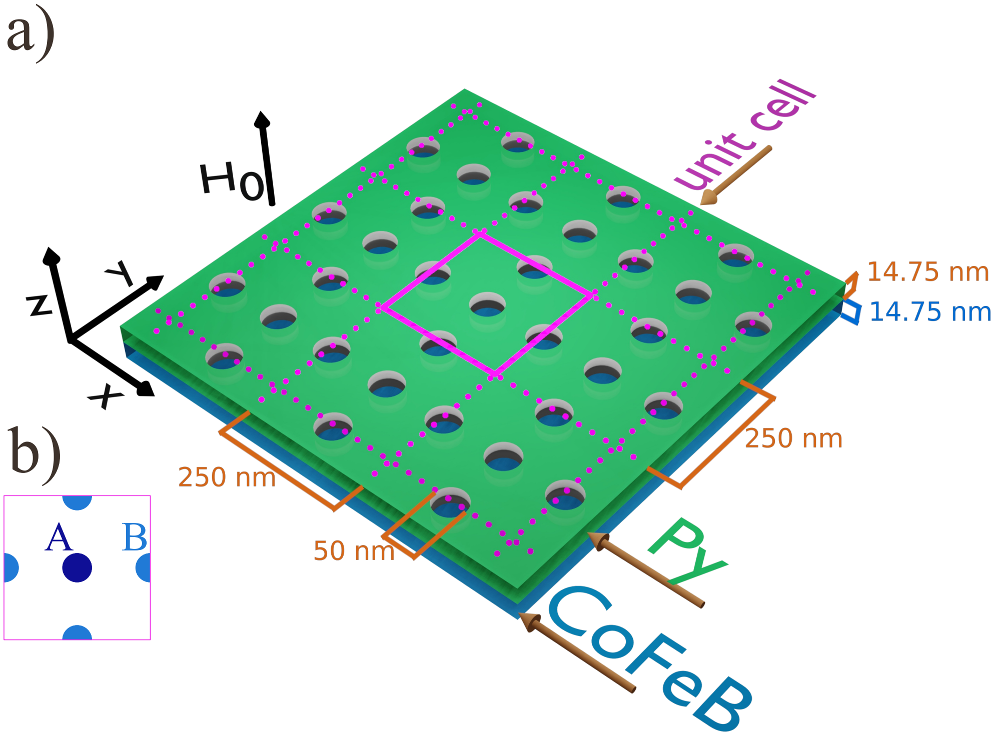

Supplementary Figure 4. Basic magnonic Lieb lattice where spin-wave excitations in the CoFeB layer are shaped by demagnetizing field from Py antidot lattice. Dimensions of the ferromagnetic unit cell are equal to 250x250x29.5 nm and contain 3 inclusions of 50 nm diameter. (a) structure of basic Lieb lattice, (b) top view on basic Lieb lattice unit cell and differentiation to nodes of sublattice A and B.

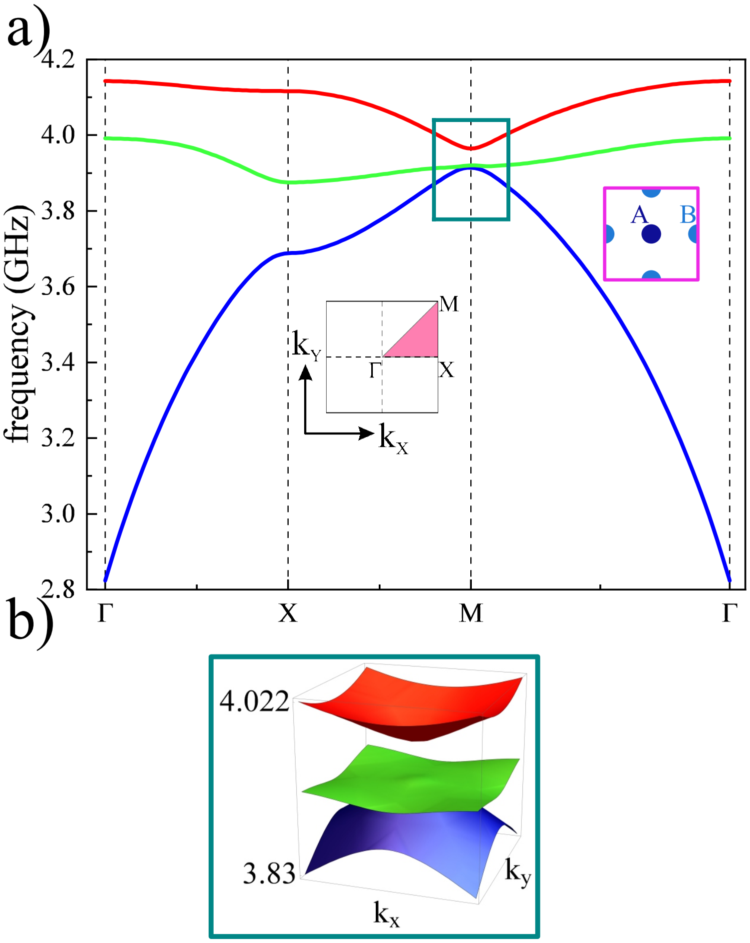

Supplementary Figure 5. The dispersion relation obtained for basic Lieb lattice formed by demagnetizing field of antidot lattice (see Supplementary Fig. 4). (a) The dispersion relation, (b) the 3D plot of dispersion relation in the region marked with the green frame in (a). Results were obtained for $H_0 = 1500$ mT applied out-of-plane.

($H_0 = 1500$ mT) to saturate the ferromagnetic material in an out-of-plane direction.

We assumed the same gyromagentic ratio for both materials $\gamma = 187$ rad T^{-1}ns^{-1}, the following values of material parameters for CoFeB [4]: saturation magnetization - M_S = 1450 mT, exchange stiffness constant - $A = 15$ pJ m^{-1}. For Py, we used material parameters [5]: saturation magnetization - $M_S = 1000$ mT, exchange stiffness constant - $A = 13$ pJ m^{-1}.

The deposition of the ADL made of Py (material of lower M_S) above the CoFeB layer (material of higher M_S) is critical for spin-wave localization in CoFeB below the exposed parts (holes) of the ADL. The demagnetization field produced on CoFeB/air interface creates

wells partially confining the spin waves. However, this pattern of internal demagnetizing field becomes smoother with increasing distance from the ADL.

The obtained dispersion relation is shown in Supplementary Fig. 5. It is worth noting that the lowest band is very dispersive, while the highest band is flattened more than in the case of the structure presented in the main part of the manuscript (see Fig. 2). The middle band, which suppose to support CLS, varies in extent similar to the third band. For this structure, Dirac cones in the M point cannot be clearly identified.

Supplementary Note 3. Lieb lattice formed by YIG inclusions in non-magnetic matrix

The periodic arrangement of ferromagnetic cylinders surrounded by non-magnetic material (e.g. air) seems to be the simplest realization of the Lieb lattice. To refer this structure to the bi-component system investigated in the main part of the manuscript, we assumed the same material and geometrical parameters for inclusions as for the structure presented in Fig. 1(a).

The advantage of this system is that the confinement of spin waves within the areas of inclusions is ensured for arbitrarily high frequency. We are not limited here by the FMR

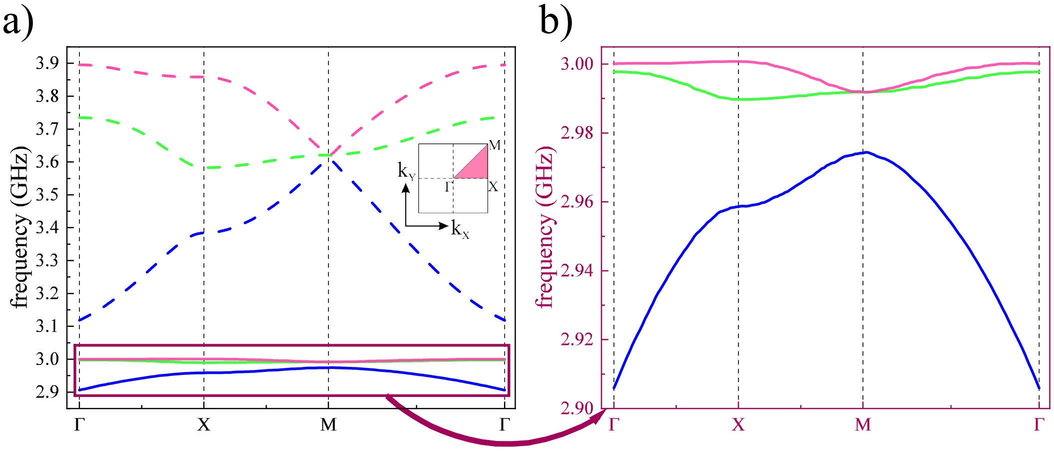

Supplementary Figure 6. Dispersion relations for basic Lieb lattice. (a) The results obtained for YIG inclusions in Ga:YIG matrix (dashed lines) and YIG inclusions without matrix (solid lines). (b) The zoomed dispersion relation obtained for YIG inclusions without matrix, marked in (a) by the frame.

frequency of the matrix, as it was for bi-component Lieb lattices (see main part of manuscript Figs. 1). However, the coupling of magnetization dynamics between the inclusions is here provided solely by the dynamical demagnetizing field, i.e. the evanescent spin waves do not participate in the coupling.

Therefore, the interaction between inclusions is much smaller in general, which leads to a significant narrowing of all magnonic bands (Supplementary Fig. 5). The widths of the second and third bands can be even smaller than the gap separating from the first bands – Supplementary Fig. 5(b). Such strong modification of the spectrum makes the applicability of the considered system for the studies of magnonic CLS questionable.

Supplementary Note 4. Demagnetizing field in YIG|Ga:YIG Lieb lattice

The difficulty in designing the magnonic system is not only due to the adjustment of geometrical parameters of the system but also due to the shaping of the internal magnetic field $\mathbf{H}_{\text{eff}}$.

The components of the effective magnetic field can be divided into long-range and short-range. The realization of our model is inseparably linked to the long-range dipolar interactions through which the coupling between inclusions is possible. This kind of interaction is sensitive to the geometry of the ferromagnetic elements forming the magnonic system.

In Lieb lattice, the nodes of minority sublattice A have four neighbors, and the nodes of majority sublattice B have two. As a result, identical inclusions (in terms of their shapes and material parameters) become distinguishable, because of slightly different values of the internal demagnetizing field. This has consequences for the formation of a frequency gap between Dirac cones at point M in the dispersion relation obtained for the basic Lieb lattice. In the literature, this phenomenon has been described for the tight-binding model and is called node dimerization of the lattice [6].

In Supplementary Fig. 7 we have shown the profile of the z-component of the demagnetizing field. For each inclusion of basic Lieb lattice through which the cut line passes, we have marked the minimum value of the demagnetizing field. The slightly lower value of internal field for inclusions A is responsible for a tiny lowering of the frequency (see Fig. 2 in the main part of manuscript) for the mode M_1 (concentrated in inclusions A) with respect to the degenerated modes M_2 and M_3 (confined in inclusions B).

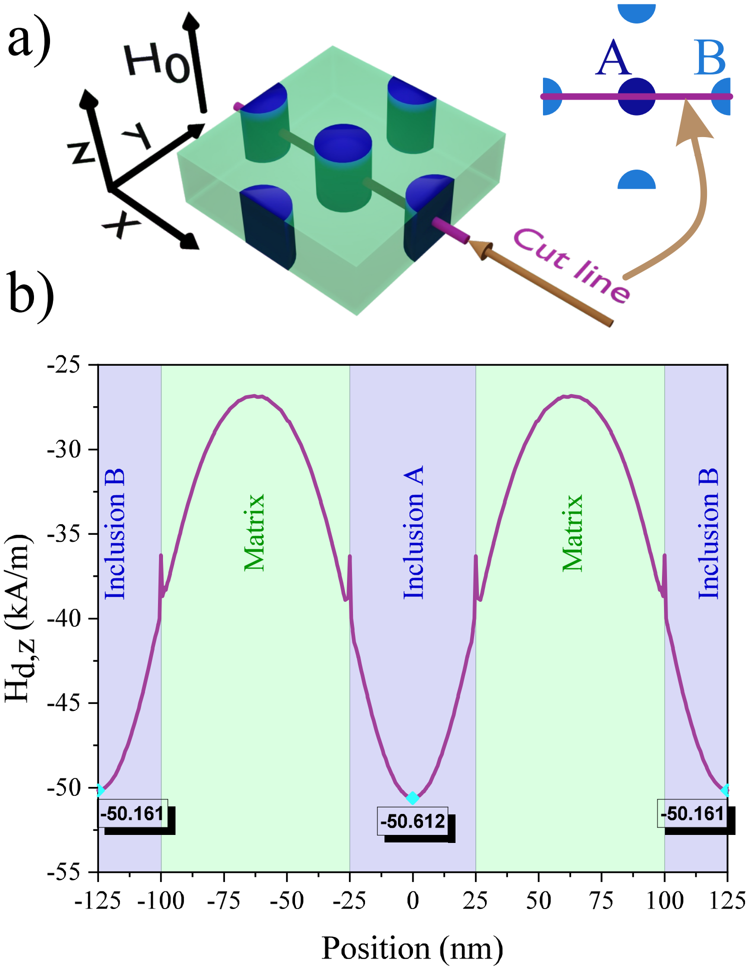

Supplementary Figure 7. Profile of static demagnetizing field plotted at a cut through (a) Lieb lattice unit cell. (b) The z-component of the demagnetizing field along the cut line is shown in (a). In the plot, we have marked peaks for the areas of inclusions A and B. Please note the slightly different values of demagnetizing in the center of A and B inclusion due to different the number of neighboring nodes: four for inclusion A, two for inclusion B.

Supplementary Note 5. Magnonic Lieb lattice – tight binding model

Let's discuss the magnetization dynamics in selected inclusion of the Lieb lattice. In the linear regime where both magnetization and effective field can be decomposed into static ($\mathbf{M}_0$ and $\mathbf{H}_{0,\text{eff}}$) and harmonically changing ($\mathbf{m}\, e^{i\omega t}$ and $\mathbf{h}_{\text{eff}}\, e^{i\omega t}$) components:

$$\mathbf{M} = \mathbf{M}_0 + \mathbf{m}\, e^{i\omega t}, \quad \mathbf{H}_{\text{eff}} = \mathbf{H}_{0,\text{eff}} + \mathbf{h}_{\text{eff}}\, e^{i\omega t}. \qquad \text{(Supp. Eq. 1)}$$

The Landau-Lifshitz equation (cf. Eq. 3 in the manuscript) takes a from, in the absence of damping ($\alpha = 0$):

$$i\omega\,\mathbf{m} = -|\gamma|\mu_0\big(\mathbf{m}\times\mathbf{H}_{0,\text{eff}} + \mathbf{M}_0\times\mathbf{h}_{\text{eff}}\big) \qquad \text{(Supp. Eq. 2)}$$

which can be expanded as:

$$\begin{cases} i\omega\,m_x = -|\gamma|\mu_0\big(m_y H_{0,\text{eff}} - M_0 h_{y,\text{eff}}\big) \\ i\omega\,m_y = -|\gamma|\mu_0\big(M_0 h_{x,\text{eff}} - m_x H_{0,\text{eff}}\big) \end{cases} \qquad \text{(Supp. Eq. 3)}$$

when $\mathbf{M}_0 = M_0\,\hat{\mathbf{z}}$ and $\mathbf{H}_{0,\text{eff}} = H_{0,\text{eff}}\,\hat{\mathbf{z}}$. By introducing the circular polarization for dynamic components: $m = m_x + im_y$ and $h = h_{x,\text{eff}} + ih_{y,\text{eff}}$, we obtain:

$$\omega\,m = |\gamma|\mu_0\big(mH_{0,\text{eff}} - M_0 h\big). \qquad \text{(Supp. Eq. 4)}$$

By expressing static effective field and saturation magnetization in the units of angular frequency: $\omega_0 = |\gamma|\mu_0 H_{0,\text{eff}}$ and $\omega_M = |\gamma|\mu_0 M_0$, we can write Supp. Eq. 4 in the form:

$$\Delta\omega\,m = -\omega_M h \qquad \text{(Supp. Eq. 5)}$$

where $\Delta\omega = (\omega - \omega_0)$ and h is the external dynamical field, resulting from the interaction with other inclusions.

According to Supp. Eq. 5, we may consider amplitudes of dynamical magnetization: m_A, m_{B_x} and m_{B_y} for the inclusions: A, B_x and B_y, respectively – see Supplementary Fig. 8. The dynamic magnetic field h perceived by inclusions A, B_x, and B_y is a linear function of the dynamic magnetization of other inclusions. In general, each inclusion is influenced by all remaining inclusions. However, following the tight-binding model, we can assume that dynamic field at each sites h_A, h_{B_x} and h_{B_y} comes from the interactions with a different set of nearest neighbors (NN). Therefore, the dynamical field perceived by each inclusion within a unit cell (see Supplementary Fig. 8) can be described by the following equations:

$$h_A = \kappa\left(\phi_x m_{B_x} + \phi_y m_{B_y}\right), \qquad \text{(Supp. Eq. 6a)}$$

$$h_{B_x} = \kappa\left(\phi_x m_A\right), \qquad \text{(Supp. Eq. 6b)}$$

$$h_{B_y} = \kappa\left(\phi_y m_A\right), \qquad \text{(Supp. Eq. 6c)}$$

where: κ is a coupling strength, $\phi_x(\mathbf{k})$ and $\phi_y(\mathbf{k})$ are the phase factor for hoppings between all NN in x- and y- directions, $\mathbf{k}$ is the wave vector $\mathbf{k} = k_x\hat{\mathbf{x}} + k_y\hat{\mathbf{y}}$. The phase factors can

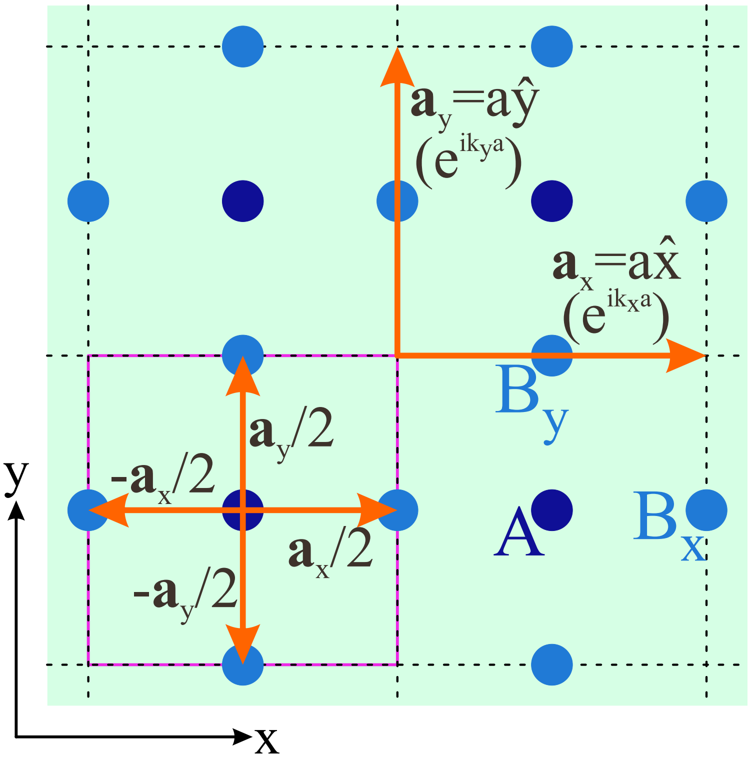

Supplementary Figure 8. Figure illustrating the basic Lieb lattice – top view. The vector $\mathbf{a}$ is lattice constant – a vector describing the transition between neighboring unit cells. Thus, $\mathbf{a}_x/2$ and $\mathbf{a}_y/2$ are the translation between the centers of neighboring inclusions in the x- and y- directions, respectively. Inclusion A is a member of the minority lattice while B_x and B_y belong to the majority lattices. Due to the periodicity of the lattice, we used Bloch-Floquet theorem and the factors $e^{ik_x a}$, $e^{ik_y a}$, related to the translation by $\mathbf{a}_x$ or $\mathbf{a}_y$ are called Floquet multipliers.

be described by the equations:

$$\phi_x(\mathbf{k}) = e^{i\mathbf{k}\mathbf{a}_x/2} + e^{i\mathbf{k}(-\mathbf{a}_x/2)} = e^{ik_x a_x/2} + e^{-ik_x a_x/2} = 2\cos\left(k_x a/2\right), \qquad \text{(Supp. Eq. 7a)}$$

$$\phi_y(\mathbf{k}) = 2\cos\left(k_y a/2\right). \qquad \text{(Supp. Eq. 7b)}$$

Taking into consideration Supp. Eq. 5 and Supp. Eq. 6, we can write:

$$\begin{cases} \Delta\omega m_A = -\omega_M \kappa \left(\phi_x m_{B_x} + \phi_y m_{B_y}\right) \\ \Delta\omega m_{B_x} = -\omega_M \kappa \phi_x m_A \\ \Delta\omega m_{B_y} = -\omega_M \kappa \phi_y m_A \end{cases} \qquad \text{(Supp. Eq. 8)}$$

in the form of algebraic eigenvalue problem:

$$\Delta\omega \begin{pmatrix} m_A \\ m_{B_x} \\ m_{B_y} \end{pmatrix} = -\kappa\omega_M \begin{pmatrix} 0 & \phi_x(\mathbf{k}) & \phi_y(\mathbf{k}) \\ \phi_x^*(\mathbf{k}) & 0 & 0 \\ \phi_y^*(\mathbf{k}) & 0 & 0 \end{pmatrix} \begin{pmatrix} m_A \\ m_{B_x} \\ m_{B_y} \end{pmatrix}. \qquad \text{(Supp. Eq. 9)}$$

By solving the eigenvalue problem Supp. Eq. 9, we obtained eigenvalues:

$$\Delta\omega = 0, \quad \Delta\omega\left(\mathbf{k}\right) = \pm 2|\omega_M\kappa|\sqrt{\cos^2\left(k_x a/2\right) + \cos^2\left(k_y a/2\right)} \qquad \text{(Supp. Eq. 10)}$$

The eigenvalue $\Delta\omega = 0$ indicates dispersion-less band, and the corresponding eigenvector [7]:

$$|m_{\mathbf{k}}\rangle = \big[\underbrace{-\cos(k_y a/2)}_{B_x}, \underbrace{0}_{A}, \underbrace{\cos(k_x a/2)}_{B_y}\big]^{\mathrm{T}}, \qquad \text{(Supp. Eq. 11)}$$

exhibits the localization on the majority sublatticies B_x, B_y, only. In the close vicinity of the M point ($\mathbf{k} = [\pi/a, \pi/a]$).

Close to the M point, we can express the dispersive bands $\Delta\omega(\mathbf{k})$ as a functions of the difference: $\mathbf{p} = \mathbf{k} - [\pi/a, \pi/a]$.

$$\Delta\omega\left(\mathbf{p}\right) = \pm 2|\omega_M\kappa|\sqrt{\cos^2\left(\left(\frac{\pi}{a} + p_x\right)\frac{a}{2}\right) + \cos^2\left(\left(\frac{\pi}{a} + p_y\right)\frac{a}{2}\right)}. \qquad \text{(Supp. Eq. 12)}$$

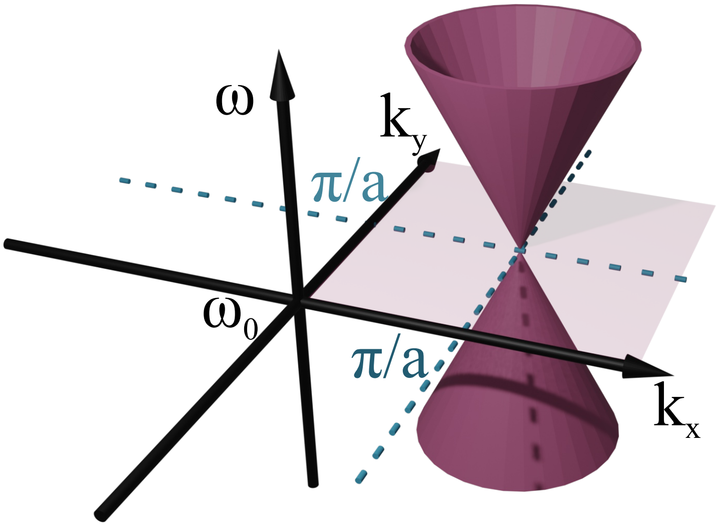

Supplementary Figure 9. Visualization of dispersion relation for basic Lieb lattice, near the M point.

For small $\mathbf{p}$, we can approximate (Supp. Eq. 12):

$$\Delta\omega\left(\mathbf{p}\right) = \pm|\omega_M\kappa|a|\mathbf{p}|, \qquad \text{(Supp. Eq. 13)}$$

where use the relation: $\cos^2\left(\frac{\pi}{2}(1+\xi)\right) \approx \left(\frac{\pi}{2}\xi\right)^2$, which is valid for small ξ. Therefore the dispersion relation for dispersive bands has a form of Dirac cones in vicinity of the M point – see Supplementary Fig. 9:

$$\omega\left(\mathbf{k}\right) = \omega_0 \pm |\omega_M\kappa|\, a \left|\mathbf{k} - \left[\frac{\pi}{a}, \frac{\pi}{a}\right]\right|. \qquad \text{(Supp. Eq. 14)}$$

---

[1] Marques, A. M. *et al.* Kaleidoscopes of hofstadter butterflies and aharonov-bohm caging from 2^n-root topology in decorated square lattices. *Phys. Rev. Res.* **5**, 023110 (2023). URL https://link.aps.org/doi/10.1103/PhysRevResearch.5.023110.

[2] Zhang, D. *et al.* New edge-centered photonic square lattices with flat bands. *Ann. Phys.* **382**, 160–169 (2017). URL https://www.sciencedirect.com/science/article/pii/S0003491617301288.

[3] Mao, X., Liu, J., Zhong, J. & Römer, R. A. Disorder effects in the two-dimensional Lieb lattice and its extensions. *Physica E Low Dimens. Syst. Nanostruct.* **124**, 114340 (2020). URL https://www.sciencedirect.com/science/article/pii/S1386947720301004.

[4] Graczyk, P. & Krawczyk, M. Coupled-mode theory for the interaction between acoustic waves and spin waves in magnonic-phononic crystals: Propagating magnetoelastic waves. *Phys. Rev. B* **96**, 024407 (2017). URL https://link.aps.org/doi/10.1103/PhysRevB.96.024407.

[5] Gallardo, R. A. *et al.* Splitting of spin-wave modes in thin films with arrays of periodic perturbations: theory and experiment. *New J. Phys.* **16**, 023015 (2014). URL https://dx.doi.org/10.1088/1367-2630/16/2/023015.

[6] Jiang, W., Huang, H. & Liu, F. A Lieb-like lattice in a covalent-organic framework and its Stoner ferromagnetism. *Nat. Commun.* **10**, 2207 (2019). URL https://www.nature.com/articles/s41467-019-10094-3.

[7] Leykam, D., Bahat-Treidel, O. & Desyatnikov, A. S. Pseudospin and nonlinear conical diffraction in Lieb lattices. *Phys. Rev. A* **86**, 031805 (2012). URL https://link.aps.org/doi/10.1103/PhysRevA.86.031805.
